# Early Mid-pregnancy Blood-Based Proteins as Possible Biomarkers of Increased Infant Birth Size in Sex-Stratified Analyses

**DOI:** 10.1007/s43032-022-01093-9

**Published:** 2022-09-30

**Authors:** Emelie Lindberger, Fredrik Ahlsson, Katja Junus, Theodora Kunovac Kallak, Susanne Lager, Paliz Nordlöf Callbo, Anna-Karin Wikström, Inger Sundström Poromaa

**Affiliations:** Department of Women’s and Children’s Health, Uppsala University, 751 85 Uppsala, Sweden

**Keywords:** Proteins, Biomarkers, Pregnancy, Birth weight

## Abstract

The objective of this study was to evaluate the associations of 92 maternal blood-based proteins with increased infant birth size. The study was performed at the Uppsala University Hospital, Sweden, and included 857 mother and child dyads. The mean age of the women was 30.3 years, and 53.2% were nulliparous. Blood samples were collected at mean 18 + 2 weeks’ gestation, and the Olink cardiovascular II panel was used to measure 92 proteins, either known to be or suspected to be markers of cardiovascular and inflammatory disease in humans. Multiple linear regression models adjusted for maternal age, parity, pre-conception BMI, height, and smoking were performed to evaluate the association of each individual protein with infant birth size. We also performed sex-stratified analyses. Eight proteins (Matrix metalloproteinase-12 (MMP-12), Prostasin (PRSS8), Adrenomedullin (ADM), Pappalysin-1 (PAPP-A), Angiotensin-converting enzyme 2 (ACE2), Sortilin (SORT1), Lectin-like oxidized LDL receptor 1 (LOX-1), and Thrombomodulin (TM)) were associated with infant birth size after false discovery rate adjustment. In the analyses including only female infants, ten proteins (MMP-12, Growth/differentiation factor 2 (GDF-2), PRSS8, SORT1, ADM, Interleukin-1 receptor antagonist protein (IL-1ra), Leptin (LEP), ACE2, TM, and Tumor necrosis factor receptor superfamily member 11A (TNFRSF11A)) were associated with infant birth size. Two proteins (PAPP-A and PRSS8) were associated with infant birth size among male infants. Our study suggests several proteins as potential biomarkers for increased birth weight, and our findings could act as a base for future research to identify new potential markers that could be added to improve screening for large infants.

**Supplementary Information:**

The online version contains supplementary material available at 10.1007/s43032-022-01093-9.

## Introduction

Excessive fetal growth and subsequently high birth weight have significant consequences on the well-being of the individual, and prediction of excessive birth weight is therefore of high clinical importance. A high birth weight (≥ 4000 g or ≥ 4500 g) [1] is associated with infant morbidity and mortality. Infants with a high birth weight run an increased risk for a complicated delivery, shoulder dystocia, admission to neonatal intensive care unit, and stillbirth [2, 3]. Long-term outcomes for individuals born with a high birth weight include obesity [4], diabetes mellitus type 1 and type 2 [4, 5], hypertension and certain malignancies in childhood, breast cancer, and several psychiatric disorders [5].

Birth weights of female and male infants differ, with males being heavier than females [6]. Since the placental function is crucial for fetal growth and development [1], sex-specific differences in its function have been proposed as underlying mechanisms behind dimorphic feto-placental growth. Male fetal-placental units are suggested to prioritize growth pathways, whereas female dittos instead invest in placental reserve capacity at the expense of a reduced growth trajectory [7]. There are also sex-specific differences in first-trimester placental biomarkers, which could indicate an effect of fetal sex on early placentation [8]. Furthermore, the impact of parental and environmental factors on fetal growth differs between sexes. For example, paternal height predicts birth length in females, whereas maternal height predicts birth length in males [9]. A high maternal birth weight has a larger impact on the birth weight of male compared with female infants [10]. In addition, prenatal maternal stress is associated with adverse birth outcomes, including reduced birth weight in female infants [11] but increased birth weight in males [12]. Hence, it is important to consider infant sex when studying effects on fetal growth and birth size.

Today, fundal height measurement is used as a screening tool for abnormal fetal growth, but the method has low sensitivity for detecting excessive growth patterns [13]. Ultrasound may also be adopted as screening method but fails to detect the majority of large infants. Universal third-trimester screening by ultrasound fetal biometry has a detection rate for LGA (birth weight > 90th percentile) of 38%, and selective screening has a detection rate of 27% [14]. Maternal blood-based biomarkers of increased infant birth size could possibly be added to current prediction models for excessive fetal growth in order to identify pregnant women with increased risk of fetal growth abnormalities.

There is a growing interest in identifying maternal blood-based biomarkers that could be used as screening tools for pregnancy complications, and some are already applied to predict clinical outcomes in obstetric care. For example, Soluble fms-like tyrosine kinase 1 (sFlt-1) and Placental growth factor (PlGF) are assessed by some centers to evaluate the likelihood of preeclampsia development [15]. In addition, measurement of Chorionic gonadotropin (hCG) and Pappalysin-1 (PAPP-A) is commonly used in first-trimester screening for chromosomal aneuploidy [16]. Measurement of maternal first-trimester circulating hCG and PAPP-A has also been evaluated in prediction models for macrosomia and large-for-gestational age (LGA) [17, 18]. Together with maternal characteristics, measurement of these proteins identify about one-third of women giving birth to macrosomic and LGA infants [17, 18].

The placenta plays an important role in fetal growth and development, and the placental function is highly dependent on adequate vascularization and regulation of the blood flow [19]. We hypothesize that proteins in the maternal circulation, related to cardiovascular and inflammatory disease, might mirror placental function and its capacity to provide the growing fetus with oxygen and nutrients. Therefore, it is of interest to study proteins associated with cardiovascular and inflammatory disease as possible biomarkers for increased birth size.

We hypothesize that maternal blood-based proteins, either known to be or suspected to be markers of cardiovascular and inflammatory disease, measured in early mid-pregnancy are associated with birth size as a proxy for fetal growth and that there might be sex-specific differences. In this study, we evaluate the associations of 92 blood-based proteins with infant birth weight in a cohort of 857 mother and child dyads. We also perform sex-stratified analyses in order to find possible sex-specific differences between female and male infants.

## Material and Methods

### Study Population

All study participants had donated a blood sample to Uppsala Biobank for Pregnant Women. Blood samples are collected to this population-based biobank in conjunction with the second-trimester anomaly scan (at 16‒20 weeks’ gestation) at Uppsala University Hospital, Uppsala, Sweden, since 2007. Women are invited to donate a blood sample if they are ≥ 18 years, Swedish-speaking, and without blood-borne disease (HIV, hepatitis C, and hepatitis B). Invitations to the Biobank are done at random, when a research nurse is available. Approximately 30% of the respondents decline participation, and the Biobank is estimated to cover about half of Uppsala County’s pregnant population [20]. After acceptance to participate and obtainment of written informed consent, a blood sample is collected, centrifuged within 2 h, and stored at − 70 °C.

In this explorative nested cohort study, we used data from two previous studies that had performed, in the same run, Olink cardiovascular II panel analyses on blood samples donated to the Biobank between 2007 and 2018. (1) Data were extracted from pregnant women without hypertension, diabetes, and renal disease selected as controls in a case–control study evaluating maternal blood-based proteins in relation to preeclampsia and small for gestation age (SGA) (*n* = 542). Data from the preeclampsia and SGA cases were not included in our study. (2) Data were extracted from pregnant women randomly enrolled from the Biobank to participate in a trial evaluating activity trackers (*n* = 315). All women included in our study had a singleton pregnancy and available data on self-reported pre-conception weight, height, and smoking habits and gave birth to a term (37 + 0 to 41 + 6 weeks) infant with registered birth weight and sex.

The study was approved by the Regional Ethical Review Board in Uppsala (Dnr: 2007/181 and Dnr: 2018/251).

### Protein Analysis

The Olink cardiovascular II panel was used to measure 92 blood-based protein biomarkers either known to be or suspected to be markers of human cardiovascular and inflammatory disease. The Olink analysis is based on a proximity assay technology developed at the Clinical Biomarkers Facility, Science for Life Laboratory, Uppsala, Sweden. Individual protein profiles are measured in the sample using antibody pairs marked with unique DNA tags. When the matched antibodies attach to the protein, their DNA tags hybridize. The hybridized DNA tags are extended to an amplicon generating a unique code for each protein. The protein profile is then read out by qPCR. The protein concentration is calculated based on the number of qPCR cycles, and the relative concentration is reported. The results are presented as Normalized Protein eXpression (NPX) values, which is an arbitrary unit in log2 scale. A high protein value corresponds to a high protein concentration [21, 22]. The full names and abbreviations of the protein biomarkers included in the Olink cardiovascular II panel are presented in Supplementary Table 1 (Online Resource).

### Covariates

Information on age, parity, pre-conception weight, height, and smoking habits was obtained from a questionnaire filled in by the woman in conjunction with the blood sampling. Body mass index (BMI) was calculated as the weight in kilograms divided by the square of the height in meters. Data on birth weight, infant sex, and gestational age at birth were extracted from the standardized pediatric electronic medical records. We calculated birth weight standard deviation scores (BWSDS) using Swedish reference standards for birth weight with respect to sex and gestational age at birth [23].

### Statistical Methods

The statistical analyses were performed using IBM SPSS Statistics version 28. A nominal two-sided *P*-value < 0.05 was considered to indicate statistical significance. Kruskal–Wallis test was performed to verify that the distribution of birth weights were the same across the different Olink batches. Multiple linear regression models were performed to evaluate the association of each individual protein with infant birth weight in grams and BWSDS. Covariates included in the model evaluating birth weight were maternal age, parity (nulliparous or parous), pre-conception BMI, height, smoking (yes or no), infant sex, and gestational age at birth. Covariates in the model evaluating BWSDS were maternal age, parity (nulliparous or parous), pre-conception BMI, height, and smoking (yes or no). To adjust for multiple testing, we used false discovery rate (FDR). In order to investigate any sex-specific differences in protein associations between sexes, we also performed the above-described analyses on female and male infants separately. To evaluate possible correlations between the proteins associated with infant birth size, Spearman’s correlations coefficients were calculated. Non-parametric tests were chosen since the proteins were not always normally distributed.

### Gene Ontology Analysis

Gene Ontology (GO) analysis was performed to identify any biological processes, besides cardiovascular and inflammatory disease, in which the proteins associated with birth size interact. The GO analysis was performed using geneontology.org on 2022–01-31 with version 10.5281/zenodo.5725227, released 2021–11-16, by using UniprotKB ID for proteins identified to be associated with birth weight in all infants, and stratified by infant sex [24, 25].

## Results

### Study Population

The study population consisted of 857 mother and child dyads with complete data. The women were between 18‒47 years of age, 456 (53.2%) were pregnant with their first child, and 322 (37.6%) were either overweight (BMI ≥ 25) or obese (BMI ≥ 30). The characteristics of the study population are presented in Table 1.

**Table 1:** Study population characteristics

Women	*N*	857
	Age, years (mean, min‒max)	30.3 (18‒47)
	Caucasian ethnicity, *n* (%)^a^	737 (96.2)
	Pre-conception BMI, kg/m^2^ (mean ± SD)	24.8 ± 4.7
	Height, cm (mean ± SD)	167 ± 6
	Nulliparous, *n* (%)	456 (53.2)
	Smoking in early mid-pregnancy, *n* (%)	23 (2.7)
	Gestational length at blood sampling, days (mean ± SD)	128 ± 11
	Asthma or allergy, *n* (%)	54 (6.3)
	Hypothyroidism or hyperthyroidism, *n* (%)	63 (7.4)
	Diabetes mellitus, *n* (%)	4 (0.5)
	Hypertension, *n* (%)	5 (0.6)
	Rheumatic disease, *n* (%)	8 (0.9)
	Inflammatory bowel disease or celiac disease, *n* (%)	14 (1.6)
	Mental illness, *n* (%)	56 (6.5)
	Condition of chronic pain, *n* (%)	10 (1.2)
	Gestational diabetes, *n* (%)	7 (0.8)
	Gestational hypertension, *n* (%)	14 (1.6)
	Preeclampsia, *n* (%)	3 (0.4)
Infants	*N*	857
	Female, *n* (%)	428 (49.9)
	Birth weight, g (mean ± SD)	3604 ± 456
	LGA, *n* (%)	27 (3.2)
	SGA, *n* (%)	5 (0.6)
	Gestational age at birth, days (mean ± SD)	279 ± 8

^a^Data on ethnicity were missing in 10.6% of the study participants

*BMI*, body mass index; *LGA*, large for gestational age (birth weight above plus two standard deviation scores of the mean birth weight for the gestational age and sex [23]); *SD*, standard deviation; *SGA*, small for gestational age (birth weight below minus two standard deviation scores of the mean birth weight for the gestational age and sex [23])

### Protein Analysis

The distribution of birth weights were the same across the different Olink batches. Since Olink recommends exclusion of assays with a large proportion of samples below the limit of detection, three proteins were excluded from further analysis: Natriuretic peptides B (BNP) (43.8% below limit of detection), Melusin (ITGB1BP2) (42.0% below limit of detection), and Carbonic anhydrase 5A, mitochondrial (CA5A) (41.4% below limit of detection). Hence, 89 protein proteins remained for analysis.

### Early Mid-pregnancy Maternal Plasma Protein Levels in Relation to Infant Birth Weight

The blood sampling was performed at mean 18 + 2 weeks’ gestation. Eight proteins were associated with both infant birth weight and BWSDS following correction for multiple testing (Table 2). Seven proteins had a positive association with these outcomes. The increase in birth weight for one unit increase in Matrix metalloproteinase-12 (MMP-12) was 131 g (95% confidence interval (CI) 85‒176 g), for Prostasin (PRSS8) 288 g (CI 165‒411 g), for Adrenomedullin (ADM) 167 g (CI 85‒250 g), for Angiotensin-converting enzyme 2 (ACE2) 99 g (CI 42‒155 g), for Sortilin (SORT1) 189 g (CI 78‒299 g), for Lectin-like oxidized LDL receptor 1 (LOX-1) 96 g (CI 35‒158 g), and for Thrombomodulin (TM) 147 g (CI 48‒245 g). PAPP-A showed a negative association with infant birth weight and BWSDS. For every unit increase in PAPP-A, birth weight decreased by 237 g (CI − 362 to − 112 g). The results for the proteins that were not associated with birth size are presented in Supplementary Table 2 (Online Resource).

**Table 2:** Associations of maternal early mid-pregnancy blood-based proteins with infant birth size (*n* = 857)

	Birth weight^a^				BWSDS^b^			
Biomarker	β	CI	*P*	*P*^*BHadj*^	β	CI	*P*	*P*^*BHadj*^
MMP-12	**131**	**85–176**	** < 0.001**	** < 0.001**	**0.29**	**0.18–0.40**	** < 0.001**	** < 0.001**
PRSS8	**288**	**165–411**	** < 0.001**	** < 0.001**	**0.70**	**0.41–0.99**	** < 0.001**	** < 0.001**
ADM	**167**	**85–250**	** < 0.001**	**0.002**	**0.40**	**0.20–0.59**	** < 0.001**	**0.002**
PAPP-A	** − 237**	** − 362 to − 112**	** < 0.001**	**0.005**	** − 0.53**	** − 0.83 to − 0.24**	** < 0.001**	**0.008**
ACE2	**99**	**42–155**	** < 0.001**	**0.011**	**0.25**	**0.11–0.38**	** < 0.001**	**0.007**
SORT1	**189**	**78–299**	** < 0.001**	**0.013**	**0.46**	**0.20–0.72**	**0.001**	**0.009**
LOX-1	**96**	**35–158**	**0.002**	**0.028**	**0.23**	**0.09–0.38**	**0.002**	**0.023**
TM	**147**	**48–245**	**0.004**	**0.040**	**0.35**	**0.12–0.58**	**0.003**	**0.039**
PlGF	**67**	**21–112**	**0.004**	**0.041**	0.15	0.04–0.26	0.007	0.054
FGF-23	53	15.1–90.7	0.006	0.054	**0.13**	**0.04–0.22**	**0.004**	**0.036**

Data are B coefficients (β) (95% confidence interval (CI)) for the change in outcome per NPX unit increase in protein concentration. Bold text indicates significant results

Data were analyzed using linear regression models

^a^Adjustments in the model for birth weight: maternal age, parity, pre-conception BMI, height, smoking in early mid-pregnancy, infant sex, and gestational age at birth

^b^Adjustments in the model for BWSDS: maternal age, parity, pre-conception BMI, height, and smoking in early mid-pregnancy

^BHadj^Benjamini-Hochberg adjusted *P*-value (raw *P*-value times number of tests divided by raw *P*-value rank)

*BMI*, body mass index; *BWSDS*, birth weight standard deviation score; *NPX*, normalized protein expression log2

### Early Mid-pregnancy Maternal Plasma Protein Levels in Relation to Birth Weight in Female and Male Infants

In female infants, ten proteins had a positive association with infant birth weight and BWSDS (Table 3). The increase in birth weight for one unit increase in MMP-12 was 170 g (CI 99‒241 g), for Growth/differentiation factor 2 (GDF-2) 144 g (CI 59‒228 g), for PRSS8 298 g (CI 125‒471 g), for SORT1 281 g (CI 127‒435 g), for ADM 200 g (CI 85‒315 g), for Interleukin-1 receptor antagonist protein (IL-1ra) 117 g (CI 43‒191 g), for Leptin (LEP) 98 g (CI 36‒160 g), for ACE2 128 g (CI 48‒208 g), for TM 210 g (CI 72‒348 g), and for Tumor necrosis factor receptor superfamily member 11A (TNFRSF11A) 155 g (CI 47‒264 g). The associations between infant birth weight and the proteins GDF-2, IL-1ra, LEP, and TNFRSF11A were significant only in the analyses including female infants. PAPP-A was not associated with female birth weight.

**Table 3:** Maternal early mid-pregnancy blood-based proteins associated with infant birth size, female and male infants analyzed separately

	Birth weight^a^				BWSDS^b^			
Biomarker	β	CI	*P*	*P*^*BHadj*^	β	CI	*P*	*P*^*BHadj*^
**Females (*n* = 428)**								
MMP-12	**170**	**99–241**	** < 0.001**	** < 0.001**	**0.40**	**0.23–0.57**	** < 0.001**	** < 0.001**
GDF-2	**144**	**59–228**	**0.001**	**0.016**	**0.35**	**0.15–0.55**	**0.001**	**0.017**
PRSS8	**298**	**125–471**	**0.001**	**0.017**	**0.75**	**0.34–1.17**	** < 0.001**	**0.017**
SORT1	**281**	**127–435**	** < 0.001**	**0.017**	**0.67**	**0.30–1.04**	** < 0.001**	**0.012**
ADM	**200**	**85–315**	**0.001**	**0.021**	**0.47**	**0.19–0.74**	**0.001**	**0.015**
IL-1ra	**117**	**43–191**	**0.002**	**0.024**	**0.30**	**0.12–0.48**	**0.001**	**0.013**
LEP	**98**	**36–160**	**0.002**	**0.025**	**0.24**	**0.09–0.39**	**0.002**	**0.018**
ACE2	**128**	**48–208**	**0.002**	**0.026**	**0.32**	**0.13–0.51**	**0.001**	**0.018**
TM	**210**	**72–348**	**0.003**	**0.030**	**0.51**	**0.18–0.84**	**0.002**	**0.024**
TNFRSF11A	**155**	**47–264**	**0.005**	**0.045**	**0.39**	**0.13–0.65**	**0.004**	**0.032**
**Males (*n* = 429)**								
PAPP-A	** − 332**	** − 506 to − 158**	** < 0.001**	**0.018**	** − 0.74**	** − 1.15 to − 0.34**	** < 0.001**	**0.033**
PRSS8	**306**	**130–481**	**0.001**	**0.020**	**0.72**	**0.31–1.13**	**0.001**	**0.026**
MMP-12	**106**	**45–166**	**0.001**	**0.028**	0.22	0.08–0.36	0.003	0.079

Data are B coefficients (β) (95% confidence interval (CI)) for the change in outcome per NPX unit increase in protein concentration. Bold text indicates significant results

Data were analyzed using linear regression models

^a^Adjustments in the model for birth weight: maternal age, parity, pre-conception BMI, height, smoking in early mid-pregnancy, and gestational age at birth

^b^Adjustments in the model for BWSDS: maternal age, parity, pre-conception BMI, height, and smoking in early mid-pregnancy

^BHadj^Benjamini-Hochberg adjusted *P*-value (raw *P*-value times number of tests divided by raw *P*-value rank)

*BMI*, body mass index; *BWSDS*, birth weight standard deviation score; *NPX*, normalized protein expression log2

**Fig. 1:**
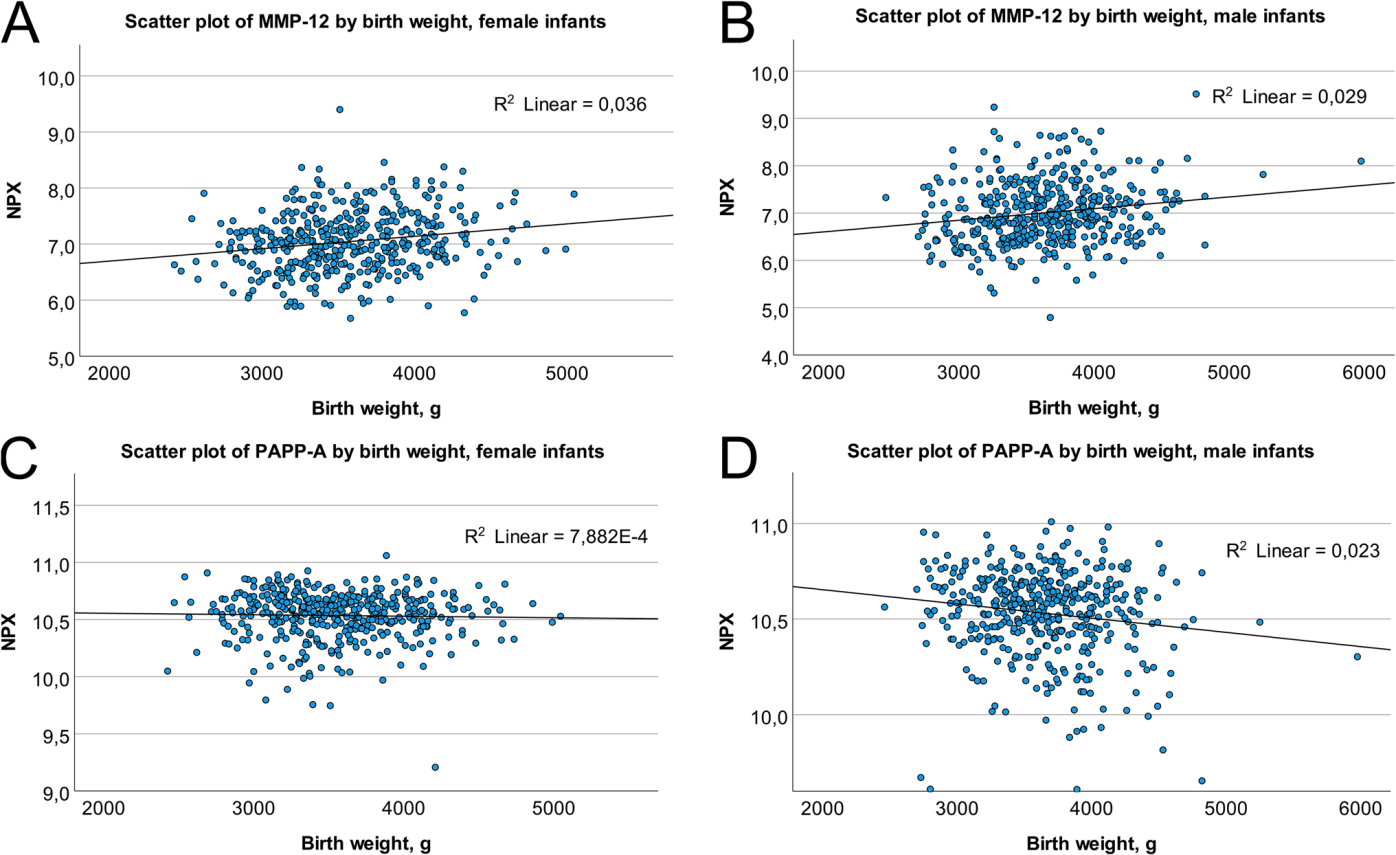
Associations of maternal early mid-pregnancy circulating levels of Matrix metalloproteinase-12 (MMP-12) and Pappalysin-1 (PAPP-A) with birth weight. There was a positive association between MMP-12 and birth weight, for both female infants (**A**) and male infants (**B**). There was no association between maternal PAPP-A levels and birth weight of female infants (**C**), but a negative association with the birth weight of male infants (**D**). The graphs include a line of best fit

**Fig. 2:**
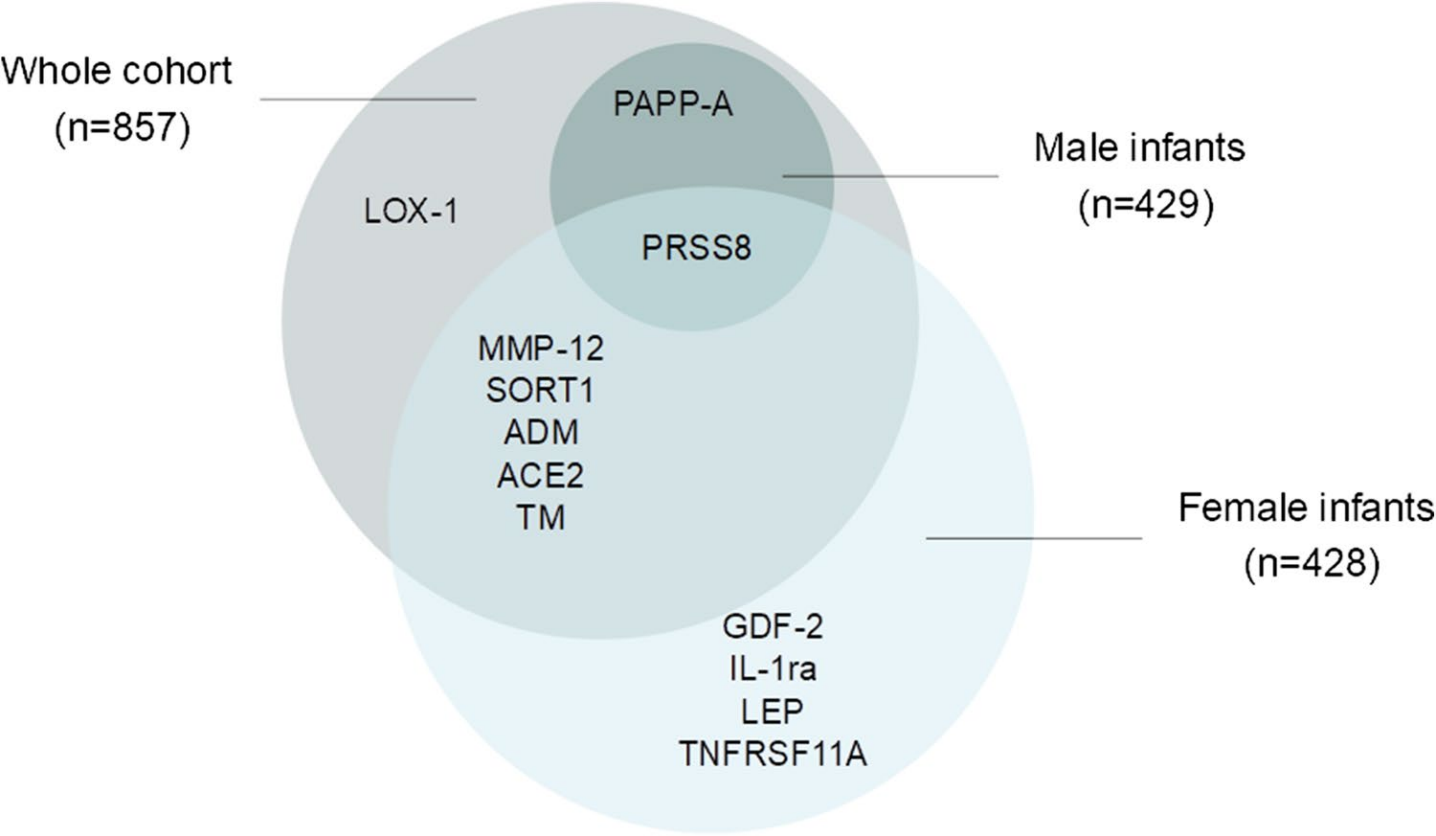
Venn diagram illustrating the distribution of proteins associated with birth weight in grams and birth weight standard deviation score in whole cohort, females, and males

In male infants, two proteins were associated with both infant birth weight and BWSDS (Table 3). PRSS8 showed a positive association with these outcomes. For every unit increase in PRSS8, birth weight increased by 306 g (CI 130‒481 g). PAPP-A had a negative association with birth weight and BWSDS. One unit increase in PAPP-A was associated with a decrease in birth weight of 332 g (CI − 506 to − 158 g). Scatter plots of MMP-12 and PAPP-A by birth weight in female and male infants are presented in Fig. 1A–D. A Venn diagram illustrating the distribution of proteins associated with birth size in the whole cohort, females, and males is presented in Fig. 2.

### Correlation Between Proteins Associated with Infant Birth Size and Gene Ontology Analysis

The Spearman’s correlation analysis showed that the proteins associated with infant birth size were correlated with each other (Supplementary Table 3, Online Resource). GO analysis for proteins identified including both sexes showed overrepresentation in GO term female pregnancy. When performed on proteins identified as associated with birth weight in female infants, GO terms within female pregnancy, cardiovascular, and inflammation were identified (Supplementary Table 4, Online Resource).

## Discussion

In this exploratory cohort study, several maternal blood-based proteins measured in early mid-pregnancy were associated with infant birth size. Our results suggest that MMP-12, PRSS8, ADM, ACE2, SORT1, LOX-1, and TM are proteins associated with increased birth weight, after adjustment for maternal age, parity, pre-conception BMI, height, and smoking habits. Our analyses also showed sex-specific differences, where more proteins were associated with birth weight among female infants than among males. Interestingly, PAPP-A was negatively associated with birth size, a finding contrary to previous studies reporting lower first-trimester PAPP-A levels among women delivering SGA infants [26].

In our analysis, MMP-12 was positively associated with birth weight. MMP-12 belongs to the matrix metalloprotease family, and MMPs are involved in the partitioning of extracellular matrix [27]. MMP-12 may play a role in tissue injury and remodeling [27]. It is suggested to be a key mediator of elastolysis when the uterine spiral arteries are remodeled in the first trimester of pregnancy [28]. Downregulation of MMP-12 gene expression has been reported in first-trimester placentas of women who later develop preeclampsia [29], but also in term placentas of obese women indicating impaired placental development [30]. Hence, increased circulating maternal MMP-12 levels might reflect appropriate placental function and adequate fetal growth.

Our results showed a positive association between PRSS8 and birth weight. The enzyme PRSS8 belongs to the serine proteinase family and cleaves peptide bonds [31]. It is found in the prostate gland, kidney, colon, lung, liver, pancreas, and salivary glands and is suggested to regulate epithelial sodium channels [32]. Mice lacking PRSS8 in the skin have a lower birth weight and die soon after birth [33]. As far as we know, PRSS8 levels in relation to birth size in humans have not previously been investigated.

Our results showed that ADM was positively associated with birth weight. ADM is a peptide hormone acting as a vasodilator; it lowers blood pressure and improves blood flow [34]. It is also suggested to preserve the integrity of the endothelial barrier [35]. Circulating ADM mRNA levels are decreased at 28 and 36 weeks’ gestation in women developing preeclampsia compared with controls [36]. Thus, elevated ADM levels might reflect appropriate placenta vascularization and function, which could explain its association with increased birth weight. Moreover, plasma ADM concentrations are higher in pregnant women with gestational diabetes mellitus (GDM), and ADM inhibits β-cell insulin production and secretion in vitro [37]. The authors outlining the above findings speculate that ADM might be partly responsible for the β-cell dysfunction seen in GDM. Gestational diabetes mellitus and increased blood glucose levels below the threshold for diabetes are associated with increased birth weight [38, 39]. Hence, the association between ADM and birth weight could possibly be explained by decreased insulin sensitivity.

We found a positive association between ACE2 and birth weight. The enzyme ACE2 is involved in the synthesis of components of the renin–angiotensin–aldosterone system and acts counter-regulatory on the renin-angiotensin axis [40]. ACE2 is expressed in human placentas and is proposed to be partly responsible for the vasoactive regulation during placentation [41]. In rats, maternal ACE2 levels increase in mid-late gestation, which might result in enhanced placental blood flow and enhanced fetal growth [42].

Our results showed a positive association between LOX-1 and birth weight. LOX-1 is a membrane receptor in vascular endothelial cells involved in recognition, internalization, and degradation of oxidized low-density lipoproteins [43]. LOX-1 is suggested to regulate trophoblast invasion and vascular remodeling in early placental development [44]. Women with preeclampsia have reduced placental LOX-1 expression compared with controls [45].

The endothelial cell membrane receptor TM showed a positive association with infant birth weight. TM has anticoagulant properties, acting as a cofactor in the thrombin-mediated activation of protein C [46]. During pregnancy, TM prevents the uteroplacental circulation from thrombotic events [47]. In pregnant women with hypertensive disorders of pregnancy, elevated plasma TM levels correlate with low birth weight [48]. The authors outlining the above findings speculate that elevated plasma TM levels might protect against the endothelial alterations of these conditions. Moreover, administration of recombinant TM attenuates fetal growth restriction in a murine model [49]. These results contrast to our results showing a positive association between TM levels and birth weight. However, we investigated a relatively healthy study cohort with a low number of new-born infants with poor growth, and the inverse relation between TM and birth weight might be restricted to women with impaired placental function.

We found a positive association between PlGF and birth weight. Highly expressed in the placenta, PlGF belongs to the vascular endothelial growth factor family and is involved in placental vascular expansion [50, 51]. Low maternal PlGF levels are associated with fetal growth restriction in women with placental dysfunction [52], which is in line with our findings that higher PlGF levels were associated with increased birth weight.

FGF-23 was positively associated with BWSDS. The growth factor FGF-23 is produced by osteocytes and regulates plasma phosphate levels [53]. FGF-23 is also suggested to be involved in innate immunity responses [54]. To the best of our knowledge, only one previous study has evaluated the relation between maternal FGF-23 levels and birth size [55]. Qamar et al. [55] report positive associations between maternal plasma FGF-23 levels at delivery and z-scores for birth length, birth weight, and head circumference. These findings are consistent with our results showing a positive association between FGF-23 and BWSDS. The authors [55] hypothesize that FGF-23 may promote fetal growth by stimulating hypertrophy and mineralization of chondrocytes, leading to intensified growth of the long bones.

The growth factor GDF-2 was positively associated with birth weight among female infants. GDF-2 is produced by the liver and maintains vascular quiescence, promotes osteogenesis, and is associated with improved glucose metabolism and insulin sensitivity [56, 57]. GDF-2 has been suggested as a biomarker for cardiovascular disease since its levels are lower in individuals with essential hypertension and coronary heart disease compared with controls [58]. In a mouse model, fetal GDF-2 levels increase until birth and remain high 3 weeks postnatal, indicating a role for GDF-2 in development [59]. To the best of our knowledge, the relation between circulating maternal GDF-2 levels and birth size has not previously been evaluated.

In our analysis, IL-1ra was positively associated with birth weight among female infants. IL-1ra is a member of the IL-1 family and binds to the IL-receptor without activating it [60]. Its biological functions are still not fully clarified, but it is suggested to be an immunomodulator [61]. IL-1ra is expressed in the endometrium [62], and is associated with favorable embryo implantation in women undergoing IVF [63]. A mouse model evaluating administered IL-1ra on the placental inflammatory response to Zika virus demonstrates preserved placental development and fetal viability [64]. Hence, IL-1 blockade by IL-1ra reduces intrauterine inflammation. One could speculate that the immunomodulatory properties of IL-1ra contribute to normal placental development and function, thereby promoting fetal growth.

In female infants, LEP was positively associated with birth weight. LEP is primarily secreted by adipocytes and regulates food intake, energy expenditure, and neuroendocrine functions [65]. It is positively correlated to body weight and percentage of body fat [66]. As pregnancy proceeds, maternal LEP levels increase [67]. A study evaluating the relations between LEP, insulin resistance, and fetal growth in 574 non-diabetic pregnancies reports associations between maternal LEP and insulin resistance, as well as maternal LEP, fetal growth, and birth size, independent of maternal BMI [68]. These results are consistent with our findings. It is assumed that LEP is involved in the mobilization of maternal fat stores in late pregnancy, leading to an increased nutrient flow to the growing fetus [69]. Thus, higher LEP levels might result in increased nutrient availability promoting fetal growth.

The transmembrane receptor TNFRSF11A was positively associated with birth weight in female infants. It is involved in the interaction between dendritic cells and T-cells and activates osteoclasts [70]. In addition, TNFRSF11A plays a role in the regulation of endometrial cell proliferation in early pregnancy, contributing to pregnancy establishment and maintenance [71]. As far as we know, no one has studied the possible mechanisms underpinning the association between TNFRSF11A and birth size.

In our analysis, PAPP-A was negatively associated with male infant birth weight. PAPP-A is a metalloprotease inactivating insulin-like growth factor (IGF) binding proteins (IGFBP), especially IGFBP-4. As IGFBP-4 inhibits IGF, PAPP-A cleavage of IGFBP-4 stimulates growth by increased IGF bioavailability [72, 73]. In normal pregnancies, PAPP-A levels increase from the first trimester until delivery [74]. Low levels of maternal first-trimester serum PAPP-A are associated with fetal aneuploidy, and PAPP-A is used as a biomarker in prenatal screening [16]. Our finding, that higher PAPP-A levels were associated with lower birth weight, is contrary to the findings reported by other investigators. A systematic review and meta-analysis reports associations between low levels of first-trimester PAPP-A and preeclampsia, preterm birth, as well as giving birth to an SGA infant [26]. In addition, others report higher first-trimester PAPP-A levels in women giving birth to macrosomic infants [17]. These contradictive results might be explained by differences in study population characteristics. In our study cohort, there were few cases of preeclampsia and SGA infants. Hence, the association between higher second-trimester PAPP-A levels and low birth weight that we found might only be representative for pregnant women with low risk for preeclampsia development and SGA. Moreover, PAPP-A is suggested as a marker of atherosclerotic disease as higher circulating PAPP-A levels are reported in individuals with coronary heart syndrome, compared with controls [75]. In addition, in a mouse model of atherosclerosis, PAPP-A-deficient mice developed lesser atherosclerotic plaques compared with wild-type mice [76]. Hence, one could speculate that increased second-trimester PAPP-A levels could reflect vessel wall atherosclerotic disease [75], which might impair placental function and, consequently, fetal growth.

Our results showed sex-specific differences; more proteins were associated with increased birth weight among female infants than among males. These dissimilarities are not easily explained since the mechanisms driving sex-specific adaptions to in utero environmental changes are poorly understood. However, previous research indicates altered placental function as well as feto-placental hormonal changes as factors that may contribute to sex-specific differences in adaption to changes in the intrauterine milieu [7].

This study has strengths and limitations. A possible limitation is that the protein concentrations are reported as relative values, which might complicate comparison with other studies using absolute protein concentrations. Nonetheless, a study comparing PlGF measured by the Olink proteomics assay with an immunochemiluminescence assay reports an excellent correlation [77].

Strengths of this study include a large, well-characterized study cohort and a considerable number of proteins analyzed by the Olink cardiovascular II panel. To the best of our knowledge, this is the first study to evaluate these proteins in relation to infant birth weight, including sex-stratified analyses. Future studies are needed to confirm our findings. It might also be of interest to evaluate the predictive value of a combination of protein levels on infant birth weight.

## Conclusion

Several maternal blood-based proteins measured in early-mid pregnancy were associated with increased infant birth size, with sex-specific differences. More proteins were associated with birth weight among female infants compared with males. Some of the identified proteins’ relation to birth size is unclear, motivating further research to explain possible causal mechanisms. Nonetheless, several of the identified proteins are related to improved vascularization and insulin sensitivity. The performance of prediction models for high birth weight is poor. Our study suggests a number of proteins as potential biomarkers for increased birth weight, and our findings could act as a base for future research to identify new possible markers that could be added to improve screening for large infants.

## Supplementary Information

Below is the link to the electronic supplementary material.

Supplementary file1 (DOCX 34 KB)

## Data Availability

The datasets generated during and/or analyzed during the current study are available from the corresponding author on reasonable request.
