# Supplementary material for: Early Mid-pregnancy Blood-Based Proteins as Possible Biomarkers of Increased Infant Birth Size in Sex-Stratified Analyses
Source: Reprod Sci. 2022 Sep 30;30(4):1165–75. doi: 10.1007/s43032-022-01093-9 (PMC9524307; doi:10.1007/s43032-022-01093-9)
Supplement: Supplementary file 1 — Supplementary file1 (DOCX 34 KB) [file 43032_2022_1093_MOESM1_ESM.docx]

**Supplementary Table 1.** List of the 92 protein biomarkers included in the Olink Cardiovacular II panel.

| **Protein name** | **Abbreviation** |
| --- | --- |
| 2,4-dienoyl-CoA reductase, mitochondrial | DECR1 |
| A disintegrin and metalloproteinase with thrombospondin motifs 13 | ADAM-TS13 |
| ADM | ADM |
| Agouti-related protein | AGRP |
| Alpha-L-iduronidase | IDUA |
| Angiopoietin-1 | ANGPT1 |
| Angiopoietin-1 receptor | TIE2 |
| Angiotensin-converting enzyme 2 | ACE2 |
| Bone morphogenetic protein 6 | BMP-6 |
| Brother of CDO | BOC |
| Carbonic anhydrase 5A, mitochondrial | CA5A |
| Carcinoembryonic antigenrelated cell adhesion molecule 8 | CEACAM8 |
| Cathepsin L1 | CTSL1 |
| C-C motif chemokine 17 | CCL17 |
| C-C motif chemokine 3 | CCL3 |
| CD40 ligand | CD40-L |
| Chymotrypsin C | CTRC |
| C-X-C motif chemokine 1 | CXCL1 |
| Decorin | DCN |
| Dickkopf-related protein 1 | Dkk-1 |
| Fatty acid-binding protein, intestinal | FABP2 |
| Fibroblast growth factor 21 | FGF-21 |
| Fibroblast growth factor 23 | FGF-23 |
| Follistatin | FS |
| Galectin-9 | Gal-9 |
| Gastric intrinsic factor | GIF |
| Gastrotropin | GT |
| Growth hormone | GH |
| Growth/differentiation factor 2 | GDF-2 |
| Heat shock 27 kDa protein | HSP 27 |
| Heme oxygenase 1 | HO-1 |
| Hydroxyacid oxidase 1 | HAOX1 |
| Interleukin-1 receptor antagonist protein | IL-1ra |
| Interleukin-1 receptor-like 2 | IL1RL2 |
| Interleukin-17D | IL-17D |
| Interleukin-18 | IL-18 |
| Interleukin-27 | IL-27 |
| Interleukin-4 receptor subunit alpha | IL-4RA |
| Interleukin-6 | IL-6 |
| Kidney injury molecule 1 | KIM1 |
| Lactoylglutathione lyase | GLO1 |
| Lectin-like oxidized LDL receptor 1 | LOX-1 |
| Leptin | LEP |
| Lipoprotein lipase | LPL |
| Low affinity immunoglobulin gamma Fc region receptor II-b | IgG Fc receptor II-b |
| Lymphotactin | XCL1 |
| Macrophage receptor MARCO | MARCO |
| Matrix metalloproteinase-12 | MMP12 |
| Matrix metalloproteinase-7 | MMP7 |
| Melusin | ITGB1BP2 |
| Natriuretic peptides B | BNP |
| NF-kappa-B essential modulator | NEMO |
| Osteoclast-associated immunoglobulinlike receptor | hOSCAR |
| Pappalysin-1 | PAPP-A |
| Pentraxin-related protein PTX3 | PTX3 |
| Placenta growth factor | PlGF |
| Platelet-derived growth factor subunit B | PDGF subunit B |
| Poly [ADP-ribose] polymerase 1 | PARP-1 |
| Polymeric immunoglobulin receptor | PIgR |
| Programmed cell death 1 ligand 2 | PD-L2 |
| Proheparin-binding EGF-like growth factor | HB-EGF |
| Pro-interleukin-16 | IL16 |
| Prolargin | PRELP |
| Prostasin | PRSS8 |
| Protein AMBP | AMBP |
| Proteinase-activated receptor 1 | PAR-1 |
| Protein-glutamine gammaglutamyltransferase 2 | TGM2 |
| Proto-oncogene tyrosine-protein kinase Src | SRC |
| P-selectin glycoprotein ligand 1 | PSGL-1 |
| Receptor for advanced glycosylation end products | RAGE |
| Renin | REN |
| Serine protease 27 | PRSS27 |
| Serine/threonine-protein kinase 4 | STK4 |
| Serpin A12 | SERPINA12 |
| SLAM family member 5 | CD84 |
| SLAM family member 7 | SLAMF7 |
| Sortilin | SORT1 |
| Spondin-2 | SPON2 |
| Stem cell factor | SCF |
| Superoxide dismutase [Mn], mitochondrial | SOD2 |
| T-cell surface glycoprotein CD4 | CD4 |
| Thrombomodulin | TM |
| Thrombopoietin | THPO |
| Thrombospondin-2 | THBS2 |
| Tissue factor | TF |
| TNF-related apoptosis-inducing ligand receptor 2 | TRAIL-R2 |
| Tumor necrosis factor receptor superfamily member 10A | TNFRSF10A |
| Tumor necrosis factor receptor superfamily member 11A | TNFRSF11A |
| Tumor necrosis factor receptor superfamily member 13B | TNFRSF13B |
| Tyrosine-protein kinase Mer | MERTK |
| Vascular endothelial growth factor D | VEGFD |
| V-set and immunoglobulin domaincontaining protein 2 | VSIG2 |

**Supplementary Table 2.** Maternal early mid-pregnancy blood-based proteins not associated with infant birth size (n=857).

|  | **Birth weight^a^** | | | | **BWSDS^b^** | | | |
| --- | --- | --- | --- | --- | --- | --- | --- | --- |
| ADAM-TS13 | 43 | -102 ‒ 187 | 0.561 | 0.861 | 0.09 | -0.25 ‒ 0.44 | 0.587 | 0.816 |
| AGRP | -36 | -102 ‒ 30 | 0.288 | 0.570 | -0.06 | -0.22 ‒ 0.09 | 0.422 | 0.683 |
| AMBP | 60 | -104 ‒ 224 | 0.474 | 0.753 | 0.21 | -0.17 ‒ 0.60 | 0.277 | 0.525 |
| ANGPT1 | 14 | -13 ‒ 40 | 0.305 | 0.578 | 0.03 | -0.03 ‒ 0.09 | 0.307 | 0.558 |
| BMP-6 | 6 | -29 ‒ 40 | 0.749 | 0.866 | 0.01 | -0.08 ‒ 0.09 | 0.881 | 0.945 |
| BOC | 79 | -36 ‒ 194 | 0.176 | 0.412 | 0.20 | -0.08 ‒ 0.47 | 0.158 | 0.352 |
| CCL17 | 9 | -22 ‒ 40 | 0.561 | 0.846 | 0.03 | -0.05 ‒ 0.10 | 0.506 | 0.763 |
| CCL3 | 33 | -18 ‒ 85 | 0.202 | 0.449 | 0.09 | -0.03 ‒ 0.21 | 0.147 | 0.354 |
| CD4 | 16 | -84 ‒ 117 | 0.750 | 0.856 | 0.04 | -0.20 ‒ 0.28 | 0.729 | 0.877 |
| CD40-L | 8 | -19 ‒ 34 | 0.576 | 0.854 | 0.02 | -0.04 ‒ 0.08 | 0.507 | 0.752 |
| CD84 | 13 | -65 ‒ 91 | 0.745 | 0.884 | 0.02 | -0.16 ‒ 0.21 | 0.805 | 0.943 |
| CEACAM8 | 48 | -6 ‒ 102 | 0.079 | 0.270 | 0.12 | -0.01 ‒ 0.24 | 0.070 | 0.231 |
| CTRC | -11 | -54 ‒ 33 | 0.627 | 0.859 | -0.02 | -0.13 ‒ 0.08 | 0.661 | 0.840 |
| CTSL1 | 83 | -15 ‒ 181 | 0.096 | 0.285 | 0.19 | -0.05 ‒ 0.42 | 0.117 | 0.306 |
| CXCL1 | 8 | -20 ‒ 35 | 0.590 | 0.847 | 0.02 | -0.05 ‒ 0.08 | 0.654 | 0.856 |
| DCN | -1 | -126 ‒ 123 | 0.983 | 0.983 | 0.02 | -0.27 ‒ 0.32 | 0.877 | 0.952 |
| DECR1 | 11 | -17 ‒ 39 | 0.448 | 0.752 | 0.03 | -0.04 ‒ 0.10 | 0.407 | 0.671 |
| Dkk-1 | 75 | 14 ‒ 135 | 0.015 | 0.097 | 0.18 | 0.04 ‒ 0.33 | 0.012 | 0.071 |
| FABP2 | -16 | -47 ‒ 14 | 0.298 | 0.577 | -0.04 | -0.11 ‒ 0.04 | 0.333 | 0.581 |
| FGF-21 | 2 | -17 ‒ 21 | 0.849 | 0.921 | 0.01 | -0.03 ‒ 0.06 | 0.593 | 0.812 |
| FS | -13 | -84 ‒ 59 | 0.721 | 0.891 | -0.01 | -0.18 ‒ 0.16 | 0.930 | 0.974 |
| Gal-9 | 45 | -45 ‒ 135 | 0.328 | 0.596 | 0.14 | -0.07 ‒ 0.35 | 0.193 | 0.409 |
| GDF-2 | 74 | 14 ‒ 133 | 0.016 | 0.094 | 0.18 | 0.04 ‒ 0.32 | 0.013 | 0.067 |
| GH | 1 | -57 ‒ 60 | 0.966 | 0.977 | 0.01 | -0.13 ‒ 0.15 | 0.861 | 0.946 |
| GIF | 9 | -30 ‒ 47 | 0.662 | 0.854 | 0.03 | -0.07 ‒ 0.12 | 0.571 | 0.807 |
| GLO1 | 36 | -10 ‒ 81 | 0.124 | 0.345 | 0.10 | 0.00 **‒** 0.21 | 0.060 | 0.214 |
| GT | -26 | -72 ‒ 20 | 0.271 | 0.561 | -0.07 | -0.18 ‒ 0.04 | 0.235 | 0.475 |
| HAOX1 | 1 | -17 ‒ 20 | 0.882 | 0.935 | 0.00 | -0.04 ‒ 0.05 | 0.846 | 0.941 |
| HB-EGF | 37 | -21 ‒ 95 | 0.208 | 0.452 | 0.09 | -0.05 ‒ 0.23 | 0.190 | 0.412 |
| HO-1 | 36 | -28 ‒ 100 | 0.274 | 0.554 | 0.08 | -0.07 ‒ 0.23 | 0.285 | 0.528 |
| hOSCAR | 58 | -62 ‒ 178 | 0.346 | 0.604 | 0.14 | -0.15 ‒ 0.42 | 0.338 | 0.579 |
| HSP 27 | -10 | -62 ‒ 43 | 0.724 | 0.883 | -0.01 | -0.14 ‒ 0.11 | 0.822 | 0.926 |
| IDUA | 44 | -17 ‒ 105 | 0.160 | 0.385 | 0.10 | -0.04 ‒ 0.25 | 0.155 | 0.354 |
| IgG Fc receptor II-b | -2 | -36 ‒ 32 | 0.920 | 0.952 | 0.00 | -0.08 ‒ 0.08 | 0.958 | 0.969 |
| IL16 | 16 | -43 ‒ 74 | 0.596 | 0.842 | 0.03 | -0.10 ‒ 0.17 | 0.627 | 0.833 |
| IL-17D | 114 | 13 ‒ 216 | 0.028 | 0.138 | 0.27 | 0.03 ‒ 0.51 | 0.028 | 0.125 |
| IL-18 | -14 | -65 ‒ 37 | 0.586 | 0.855 | -0.02 | -0.14 ‒ 0.11 | 0.812 | 0.927 |
| IL-1ra | 55 | 3 ‒ 107 | 0.039 | 0.165 | 0.15 | 0.03 ‒ 0.28 | 0.014 | 0.071 |
| IL1RL2 | 62 | -1 ‒ 125 | 0.055 | 0.213 | 0.15 | 0.00 **‒** 0.30 | 0.054 | 0.209 |
| IL-27 | 16 | -72 ‒ 104 | 0.728 | 0.876 | 0.04 | -0.17 ‒ 0.25 | 0.702 | 0.868 |
| IL-4RA | 94 | -5 ‒ 193 | 0.063 | 0.234 | 0.22 | -0.01 ‒ 0.46 | 0.063 | 0.216 |
| IL-6 | 48 | 4 ‒ 92 | 0.033 | 0.155 | 0.13 | 0.03 ‒ 0.24 | 0.012 | 0.074 |
| KIM1 | 44 | -7 ‒ 95 | 0.094 | 0.288 | 0.11 | -0.01 ‒ 0.23 | 0.073 | 0.232 |
| LEP | 44 | 0 ‒ 89 | 0.052 | 0.210 | 0.11 | 0.01 ‒ 0.22 | 0.037 | 0.157 |
| LPL | -22 | -83 ‒ 39 | 0.473 | 0.765 | -0.08 | -0.22 ‒ 0.06 | 0.274 | 0.530 |
| MARCO | 152 | 39 ‒ 265 | 0.009 | 0.064 | 0.38 | 0.11 ‒ 0.64 | 0.006 | 0.054 |
| MERTK | 72 | -9 ‒ 153 | 0.080 | 0.264 | 0.16 | -0.04 ‒ 0.35 | 0.111 | 0.309 |
| MMP7 | -6 | -75 ‒ 64 | 0.871 | 0.934 | 0.00 | -0.17 ‒ 0.16 | 0.980 | 0.980 |
| NEMO | 7 | -24 ‒ 37 | 0.673 | 0.844 | 0.02 | -0.05 ‒ 0.09 | 0.595 | 0.802 |
| PAR-1 | 14 | -51 ‒ 80 | 0.666 | 0.847 | 0.03 | -0.12 ‒ 0.19 | 0.677 | 0.849 |
| PARP-1 | 59 | 4 ‒ 114 | 0.034 | 0.153 | 0.13 | 0.00 ‒ 0.26 | 0.046 | 0.186 |
| PDGF subunit B | 20 | -7 ‒ 47 | 0.152 | 0.376 | 0.05 | -0.02 ‒ 0.11 | 0.153 | 0.358 |
| PD-L2 | 117 | 30 ‒ 204 | 0.008 | 0.069 | 0.27 | 0.07 ‒ 0.48 | 0.009 | 0.060 |
| PIgR | 57 | -163 ‒ 277 | 0.613 | 0.852 | 0.18 | -0.34 ‒ 0.70 | 0.493 | 0.757 |
| PRELP | -6 | -162 ‒ 150 | 0.941 | 0.963 | 0.01 | -0.36 ‒ 0.38 | 0.944 | 0.966 |
| PRSS27 | 8 | -53 ‒ 68 | 0.806 | 0.897 | 0.01 | -0.14 ‒ 0.15 | 0.932 | 0.965 |
| PSGL-1 | 9 | -117 ‒ 135 | 0.890 | 0.932 | 0.09 | -0.21 ‒ 0.39 | 0.556 | 0.811 |
| PTX3 | 19 | -63 ‒ 100 | 0.652 | 0.866 | 0.06 | -0.13 ‒ 0.26 | 0.660 | 0.851 |
| RAGE | -17 | -95 ‒ 60 | 0.661 | 0.865 | -0.05 | -0.24 ‒ 0.13 | 0.563 | 0.808 |
| REN | 84 | 18 ‒ 149 | 0.012 | 0.085 | 0.21 | 0.05 ‒ 0.36 | 0.008 | 0.062 |
| SCF | 61 | -15 ‒ 136 | 0.114 | 0.327 | 0.15 | -0.03 ‒ 0.33 | 0.103 | 0.296 |
| SERPINA12 | 11 | -17 ‒ 39 | 0.451 | 0.743 | 0.03 | -0.04 ‒ 0.09 | 0.443 | 0.704 |
| SLAMF7 | 23 | -24 ‒ 71 | 0.339 | 0.603 | 0.05 | -0.06 ‒ 0.16 | 0.392 | 0.658 |
| SOD2 | -34 | -245 ‒ 176 | 0.748 | 0.876 | -0.04 | -0.53 ‒ 0.46 | 0.887 | 0.940 |
| SPON2 | 153 | -16 ‒ 321 | 0.076 | 0.271 | 0.39 | -0.01 ‒ 0.78 | 0.058 | 0.215 |
| SRC | 3 | -24 ‒ 29 | 0.835 | 0.917 | 0.01 | -0.05 ‒ 0.07 | 0.748 | 0.888 |
| STK4 | -4 | -28 ‒ 21 | 0.756 | 0.852 | -0.01 | -0.07 ‒ 0.05 | 0.810 | 0.936 |
| TF | 43 | -63 ‒ 149 | 0.425 | 0.727 | 0.13 | -0.12 ‒ 0.38 | 0.315 | 0.561 |
| TGM2 | 42 | -13 ‒ 97 | 0.136 | 0.367 | 0.12 | -0.01 ‒ 0.25 | 0.075 | 0.230 |
| THBS2 | 105 | -55 ‒ 266 | 0.198 | 0.452 | 0.29 | -0.09 ‒ 0.67 | 0.135 | 0.343 |
| THPO | 43 | -42 ‒ 128 | 0.322 | 0.597 | 0.13 | -0.07 ‒ 0.33 | 0.195 | 0.404 |
| TIE2 | 82 | -28 ‒ 192 | 0.143 | 0.364 | 0.21 | -0.05 ‒ 0.47 | 0.112 | 0.302 |
| TNFRSF10A | 105 | 19 ‒ 192 | 0.017 | 0.097 | 0.24 | 0.04 ‒ 0.45 | 0.020 | 0.093 |
| TNFRSF11A | 95 | 13 ‒ 177 | 0.023 | 0.119 | 0.25 | 0.05 ‒ 0.44 | 0.013 | 0.070 |
| TNFRSF13B | 69 | -9 ‒ 147 | 0.083 | 0.264 | 0.16 | -0.02 ‒ 0.35 | 0.086 | 0.255 |
| TRAIL-R2 | 52 | -16 ‒ 119 | 0.136 | 0.356 | 0.12 | -0.04 ‒ 0.28 | 0.142 | 0.351 |
| VEGFD | -16 | -83 ‒ 52 | 0.651 | 0.878 | -0.03 | -0.19 ‒ 0.13 | 0.725 | 0.884 |
| VSIG2 | 20 | -47 ‒ 88 | 0.552 | 0.862 | 0.06 | -0.10 ‒ 0.22 | 0.449 | 0.701 |
| XCL1 | -32 | -84 ‒ 21 | 0.236 | 0.500 | -0.07 | -0.20 ‒ 0.05 | 0.256 | 0.506 |

Data are B coefficients (β) (95% confidence interval (CI)) for the change in outcome per NPX unit increase in protein concentration.

Data were analyzed using linear regression models.

^a^ Adjustments in the model for birth weight: maternal age, parity, pre-conception BMI, height, smoking in early mid-pregnancy, infant sex, and gestational age at birth.

^b^ Adjustments in the model for BWSDS: maternal age, parity, pre-conception BMI, height, and smoking in early mid-pregnancy.

^BHadj^ Benjamini-Hochberg adjusted *P*-value (raw *P*-value times number of tests divided by raw *P*-value rank)

BMI, body mass index; BWSDS, birth weight standard deviation score; NPX, normalized protein expression log2

|  | **ADM** | **ACE2** | **FGF-23** | **GDF-2** | **IL-1ra** | **LEP** | **LOX-1** | **MMP12** | **PAPP-A** | **PlGF** | **PRSS8** | **SORT1** | **TM** |
| --- | --- | --- | --- | --- | --- | --- | --- | --- | --- | --- | --- | --- | --- |
| **ACE2** | 0.19^***^ |  |  |  |  |  |  |  |  |  |  |  |  |
| **FGF-23** | 0.24^***^ | 0.11^**^ |  |  |  |  |  |  |  |  |  |  |  |
| **GDF-2** | 0.04 | 0.21^***^ | 0.04 |  |  |  |  |  |  |  |  |  |  |
| **IL-1ra** | 0.18^***^ | 0.11^**^ | 0.19^***^ | -0.04 |  |  |  |  |  |  |  |  |  |
| **LEP** | 0.08^*^ | 0.03 | 0.15^***^ | -0.04 | 0.42^***^ |  |  |  |  |  |  |  |  |
| **LOX-1** | 0.22^***^ | 0.18^***^ | 0.13^***^ | 0.12^**^ | 0.31^***^ | 0.04 |  |  |  |  |  |  |  |
| **MMP12** | 0.07^*^ | 0.04 | 0.07^*^ | 0.15^***^ | -0.04 | -0.11^**^ | 0.09^**^ |  |  |  |  |  |  |
| **PAPP-A** | 0.05 | -0.02 | 0.04 | -0.12^***^ | 0.16^***^ | 0.18^***^ | -0.08^*^ | -0.20^***^ |  |  |  |  |  |
| **PlGF** | 0.40^***^ | 0.15^***^ | 0.12^***^ | 0.12^***^ | 0.03 | -0.09^**^ | 0.18^***^ | 0.10^**^ | -0.09^**^ |  |  |  |  |
| **PRSS8** | 0.50^***^ | 0.29^***^ | 0.22^***^ | 0.24^***^ | 0.22^***^ | 0.13^***^ | 0.26^***^ | 0.13^***^ | -0.07 | 0.32^***^ |  |  |  |
| **SORT1** | 0.23^***^ | 0.27^***^ | 0.27^***^ | 0.34^***^ | 0.16^***^ | 0.08^*^ | 0.18^***^ | 0.20^***^ | -0.06 | 0.11^**^ | 0.29^***^ |  |  |
| **TM** | 0.31^***^ | 0.18^***^ | 0.26^***^ | 0.19^***^ | 0.22^***^ | 0.12^***^ | 0.20^***^ | 0.11^**^ | 0.03 | 0.11^**^ | 0.43^***^ | 0.33^***^ |  |
| **TNFRSF11A** | 0.44^***^ | 0.18^***^ | 0.23^***^ | 0.09^*^ | 0.40^***^ | 0.29^***^ | 0.23^***^ | 0.03 | 0.09^**^ | 0.09^*^ | 0.49^***^ | 0.29^***^ | 0.47^***^ |

**Supplementary Table 3.** Spearman’s correlation coefficients for the associations between proteins associated with birth size.

**P* <0.05, ** *P* <0.01, *** *P* <0.001

**Supplementary Table 4.** Results of Gene Ontology analysis on proteins associated with birth size in female infants.

| **Gene Ontology Biological processes** | **Fold Enrichment** | ***P*** | ***P*^adj^** |
| --- | --- | --- | --- |
| Positive regulation of multicellular organismal process (GO:0051240) | 11.54 | <0.001 | <0.001 |
| Response to organic substance (GO:0010033) | 6.20 | <0.001 | 0.007 |
| Multi-multicellular organism process (GO:0044706) | 41.19 | <0.001 | 0.007 |
| Regulation of multicellular organismal process (GO:0051239) | 6.29 | <0.001 | 0.008 |
| Female pregnancy (GO:0007565) | 48.18 | <0.001 | 0.008 |
| Regulation of vasoconstriction (GO:0019229) | 98.07 | <0.001 | 0.008 |
| Ossification (GO:0001503) | 29.74 | <0.001 | 0.009 |
| Positive regulation of ion transport (GO:0043270) | 29.96 | <0.001 | 0.009 |
| Regulation of response to external stimulus (GO:0032101) | 12.97 | <0.001 | 0.009 |
| Regulation of defense response (GO:0031347) | 16.40 | <0.001 | 0.009 |
| Elastin metabolic process (GO:0051541) | > 100 | <0.001 | 0.010 |
| Regulation of organic acid transport (GO:0032890) | 87.02 | <0.001 | 0.010 |
| Negative regulation of vasoconstriction (GO:0045906) | > 100 | <0.001 | 0.012 |
| Regulation of cytokine production (GO:0001817) | 14.13 | <0.001 | 0.012 |
| Regulation of anion transport (GO:0044070) | 67.90 | <0.001 | 0.012 |
| Regulation of response to stress (GO:0080134) | 9.38 | <0.001 | 0.012 |
| Response to oxygen-containing compound (GO:1901700) | 8.19 | <0.001 | 0.023 |
| Positive regulation of cell population proliferation (GO:0008284) | 11.14 | <0.001 | 0.027 |
| Response to organonitrogen compound (GO:0010243) | 10.92 | <0.001 | 0.028 |
| Regulation of tube size (GO:0035150) | 44.13 | <0.001 | 0.028 |
| Regulation of cell population proliferation (GO:0042127) | 7.51 | <0.001 | 0.028 |
| Regulation of tube diameter (GO:0035296) | 44.45 | <0.001 | 0.029 |
| Blood vessel diameter maintenance (GO:0097746) | 44.45 | <0.001 | 0.031 |
| Response to peptide (GO:1901652) | 19.29 | <0.001 | 0.031 |
| Regulation of response to stimulus (GO:0048583) | 4.21 | <0.001 | 0.032 |
| Positive regulation of cytokine production (GO:0001819) | 17.49 | <0.001 | 0.033 |
| Response to nitrogen compound (GO:1901698) | 9.98 | <0.001 | 0.037 |
| Response to chemical (GO:0042221) | 4.12 | <0.001 | 0.037 |
| Regulation of blood pressure (GO:0008217) | 33.40 | <0.001 | 0.046 |

The Gene Ontology analysis was performed by use of geneontology.org with UniprotKB ID for proteins.

^adj^ FDR-adjusted *P*-value

GO, Gene Ontology; FDR, false discovery rate
